# An overview of the laboratory diagnosis of *Pneumocystis jirovecii* pneumonia

**DOI:** 10.1128/jcm.00361-24

**Published:** 2025-02-03

**Authors:** Alexis Jaramillo Cartagena, Osaretin Emmanuel Asowata, Dianna Ng, N. Esther Babady

**Affiliations:** 1 Clinical Microbiology Service, Department of Pathology and Laboratory Medicine, Memorial Sloan Kettering Cancer Center 5803 , New York, New York, USA; 2 Cytology Service, Department of Pathology and Laboratory Medicine, Memorial Sloan Kettering Cancer Center 5803 , New York, New York, USA; 3 Infectious Disease Service, Department of Medicine, Memorial Sloan Kettering Cancer Center 5803 , New York, New York, USA; Vanderbilt University Medical Center, Nashville, Tennessee, USA

**Keywords:** *Pneumocystis*, stains, immunofluorescent, PCR, immunocompromised hosts

## Abstract

*Pneumocystis jirovecii* (*P. jirovecii*) is a fungal pathogen associated with significant morbidity in immunocompromised patients, including both HIV- and non-HIV-infected patients. The nonspecific clinical and radiological presentation makes clinical diagnostic challenging, emphasizing the need for accurate laboratory diagnostic tests. However, *P. jirovecii* does not grow in routine culture media, which presents diagnostic challenges in the laboratory as well. Recent publications from the European Organization for Research and Treatment of Cancer and the Mycoses Study Group Education and Research Consortium continue to rely on direct detection of *P. jirovecii* organisms in tissues and respiratory samples to define proven *P. jirovecii* pneumonia (PCP) even as the sensitivity of these methods are lower. Novel, standardized methods are needed to improve the clinical and laboratory diagnosis and management of PCP. This minireview provides an overview of current diagnostic tests for PCP and emerging applications that aim at filling existing diagnostic gaps and providing more accurate and less invasive diagnoses for this significant disease.

## INTRODUCTION

*Pneumocystis jirovecii*, formerly known as *Pneumocystis carinii* (1), is an opportunistic fungal pathogen, first identified by Carlos Chagas in Brazil in 1909. Chagas studied the protozoa *Trypanosoma cruzi* and mistakenly identified *Pneumocystis* cysts as a schizogonic form of *T. cruzi* in lung sections of guinea pigs (2). In 1912, a couple at the Pasteur Institute in France, Delanoe and Delanoe observed similar structures in *Trypanosoma*-free rats and proposed the name *Pneumocystis carinii*, deducing that they represented a new organism (3, 4). The organism was named *Pneumocystis carinii* in honor of Antonio Carini, director of the Pasteur Institute.

*P. carinii* was initially classified as a protozoan, amidst intense debate, as the two unique forms of this organism, the ascus (cyst) and the trophic (trophozoite) life forms, resembled stages observed in a typical protozoa life cycle (5–7). The name of the human species, *Pneumocystis jirovecii,* was coined in 1976 in honor of Otto Jirovec, a Czechoslovak parasitologist and protozoologist, who linked this organism to epidemics of interstitial plasma cell pneumonia in neonates in Europe (2). *P. jirovecii* was ultimately reclassified as a member of the ascomycetous fungi Kingdom in 1988, after sequencing data showed homology with other fungal organisms (4, 8).

*P. jirovecii* causes severe pneumonia, primarily in immunocompromised patients. The disease caused by *P. jirovecii* was originally referred to as PCP (*Pneumocystis carinii* pneumonia) in relation to its prior name. Although PJP (*Pneumocystis jirovecii* pneumonia) is often used, the abbreviation PCP has persisted, standing for *Pneumocystis jirovecii* pneumonia.

In this minireview, we briefly review the epidemiology and clinical presentation of PCP and describe current and emerging laboratory diagnostics for *Pneumocystis jirovecii*.

## TRANSMISSION AND EPIDEMIOLOGY

The mode of transmission of *P. jirovecii* is not fully understood due in part to the difficulty of culturing the organism. The potential for human-to-human transmission of *P. jirovecii* and the existence of an environmental reservoir have been studied extensively. Immunocompromised and immunocompetent mouse models showed that *P. jirovecii* can be transmitted to each other when in proximity (9), and co-housing studies of immunocompromised animal models have shown that the ascus is the environmental and transmissible form (10, 11). The trophic form is released after the cysts are inhaled into the lungs where they adhere to type 1 epithelium to establish infection (7, 11, 12). The trophic form can potentially replicate sexually and asexually to form new cysts to potentially continue the infection cycle (13). Although it is hard to extrapolate the mouse model result to humans, given the difference in *Pneumocystis* species, a similar result was reported in human renal transplant patients (14). In addition, *P. jirovecii* DNA was detected in the air around a patient with a confirmed case of PCP (15, 16). *P. jirovecii* was reportedly detected in water and soil as well; thus, people who participate in camping, hiking, and gardening are most at risk (17). Host colonization by *P. jirovecii* has been reported, and these colonized individuals may serve as potential reservoirs for future transmission (18). Exposure to *P. jirovecii* is hypothesized to occur in early childhood through inhalation of the ascus. This hypothesis is supported by the detection of *P. jirovecii* antibodies in 83% of children by 7 months of age (19). Reactivation may occur later in life due to diseases and conditions such as HIV, solid organ or stem cell transplant, and prolonged glucocorticoid use, which may increase the risk for pneumocystis pneumonia (20).

In the pre-Human immunodeficiency virus/acquired immunodeficiency syndrome (HIV/AIDS) epidemic era, PCP was a relatively rare infection seen primarily in pediatric oncology patients and malnourished people (19). In the early 1980s, PCP became an AIDS-defining illness following reports of *P. jirovecii* detection and associated high mortality among AIDS patients (21). The greatest risk of PCP was among people living with HIV with CD4 T cell counts below 200 cells/mm^3^ (22, 23). The development of highly active antiretroviral therapy (HAART) and trimethoprim-sulfamethoxazole (TMP/SMX) has greatly decreased cases of PCP among HIV/AIDS-infected patients, especially in developed countries (24, 25). PCP is now increasingly seen in immunosuppressed, non-HIV-infected individuals including those with primary immune deficiencies, autoimmune conditions with long-term steroid use, hematological malignancies, solid organ transplants, inflammatory diseases, patients on anti-tumor treatments, and patients on immunosuppressive drugs (26–29).

## CLINICAL AND RADIOLOGICAL PRESENTATION

PCP is difficult to diagnose clinically and radiologically as symptoms are non-specific. PCP commonly presents with persistent or intermittent fever, progressive dyspnea, hypoxemia, a non-productive cough, chest discomfort, fatigue, and weight loss (7, 25, 30, 31). In severe cases, PCP can progress to respiratory failure (7). Patients with HIV are more likely to exhibit asymptomatic PCP infection. In one meta-analysis study looking at the prevalence of PCP in HIV-positive patients in Africa, PCP was detected in 9% of 140 asymptomatic adult patients, compared with 19% of 3,583 individuals with respiratory symptoms (32). When symptomatic, respiratory distress may be mild or moderate with symptoms typically present for 1–2 weeks before presentation (33, 34). In contrast, HIV-negative individuals present with a more sudden onset of symptoms and a more severe clinical presentation (34–37). Immunocompromised individuals without appropriate prophylaxis, such as TMP/SMX, are at an increased risk of developing PCP (25). Mortality can be high, ranging from 7% to 20% in HIV-positive patients and 29% to 60% in HIV-negative patients (7, 27, 37–41).

Extrapulmonary pneumocystis (EPCP), a rare complication of PCP, has been identified in various tissues including lymph nodes, thymus, liver, spinal column, etc. (42–44). Prior to the use of HAART, EPCP occurred primarily in patients with AIDS. EPCP has also been reported in other immunocompromised patients including patients with hematologic malignancies and transplant patients, both solid organ and hematopoietic stem cell recipients (42).

PCP classically presents with bilateral, diffuse interstitial infiltrates, which appear as a fine reticular pattern across the lung fields and are frequently described as having a “ground-glass” appearance on chest X-rays (45). This radiographic feature is indicative of alveolar filling due to the presence of the organism and an accompanying inflammatory response. However, chest X-rays can occasionally appear normal, especially during the early stages of the disease (25). More recently, high-resolution computed tomography (HRCT) scans have demonstrated higher sensitivity than standard chest X-rays, often revealing more prominent ground-glass opacities that suggest significant alveolar involvement (46). Lung ultrasound has emerged as a useful point-of-care tool, particularly in resource-limited settings where X-rays and CT scans may not be readily available; it can detect pulmonary comet-tail artifacts, which may correspond to the ground-glass opacities observed on CT and X-rays (47).

## LABORATORY TESTING

*P. jirovecii* does not grow on routine mycology media, making diagnosis and research into novel treatment strategies for this organism challenging. Current laboratory methods for the detection of *P. jirovecii* include microscopy, nucleic acids amplification tests, and antigen and antibody testing.

### Specimen types for direct testing

Routine diagnosis of PCP is made using lower respiratory tract secretions including induced sputum and bronchoalveolar lavage (BAL) fluids (48). BAL fluid is preferred over induced sputum as it provides a high-quality sample directly from the lungs without the need for a more invasive lung biopsy (49–53). However, as diagnostic bronchoscopy is still an invasive procedure, collection of BAL fluids may not always be possible, particularly in thrombocytopenic patients due to an increased risk for bleeding. PCP diagnosis can be made using less-invasive specimens like induced sputum (54), nasopharyngeal aspirate (55), and oral washings (56–59). When possible, the collection of lung biopsies is most valuable for diagnosing PCP (60). Histological examination of lung biopsy samples allows for the visualization of PCP through specialized stains that highlight the organism forms within the tissue. Other specimens that have been used include pleural fluid (61) and ascetic fluid (62).

### Direct detection by microscopy

**Fig 1:**
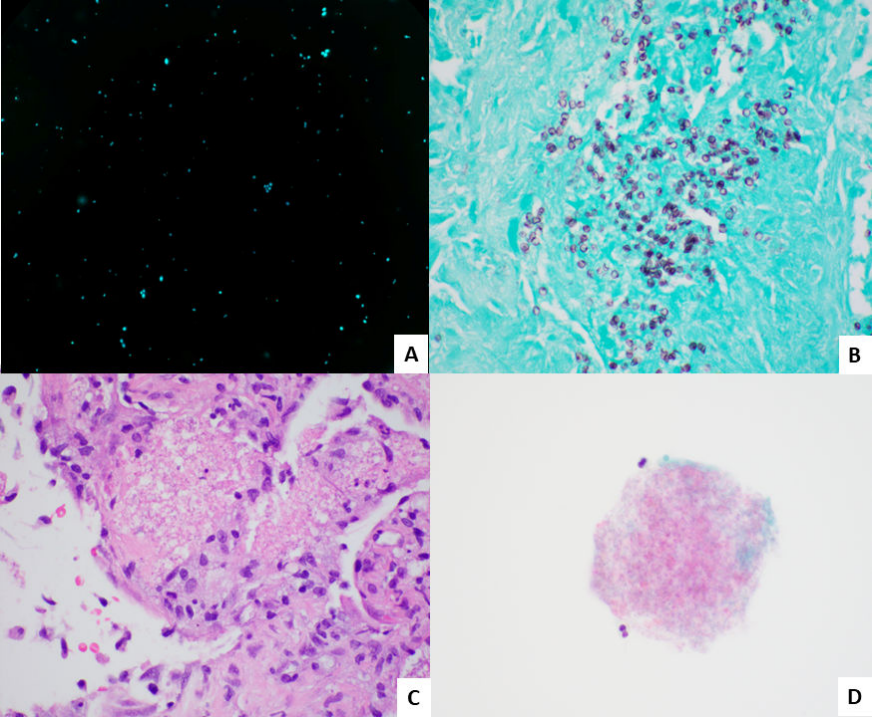
Select stains for PCP in clinical samples. (**A**) KOH-Calcofluor white stain of a bronchoalveolar lavage showing small cyst forms of *P. jirovecii* (400×). (**B**). Grocott’s methenamine silver-stained section of a lung biopsy highlighting thick-walled ovoid and crescentic yeast, some with central dot-like densities (600×). (**C**) Hematoxylin and eosin-stained section of a lung biopsy showing necrotizing granulomatous inflammation and a frothy intraalveolar eosinophilic exudate (600×). (**D**) ThinPrep slide (Papanicolaou stain) of a bronchoalveolar lavage showing a foamy aggregate with small, empty spaces (600×).

PCP can be diagnosed by direct visualization of the characteristic ascus/cyst (size range: 5–8 µm) or trophic forms (size range: 1–4 µm) through microscopy by personnel with the technical expertise using various stains, each suited for different specimen types, such as lung biopsies or respiratory secretions (63–67). The sensitivity of microscopic methods is hampered by the lower fungal load especially among non-HIV-infected patients compared with those with HIV infection (68). Each stain has unique advantages and limitations related to sensitivity, specificity, speed, complexity, cost, and institutional resources (63, 69). These factors are particularly important in clinical settings, where timely diagnosis can significantly impact patient outcomes. Examples of stained samples are shown in Fig. 1A through E.

In clinical microbiology laboratories, the most used stains for respiratory secretions are Calcofluor white, Giemsa-like stains, and immunofluorescent assays. Other stains, such as hematoxylin and eosin, periodic Acid-Schiff, Papanicolaou’s, and Grocott’s Methenamine Silver, are more routinely performed in anatomical pathology laboratories, particularly those specializing in cytology-based assays. Molecular testing is typically conducted at reference laboratories or in laboratories with advanced infrastructure.

- **Calcofluor White (CW):** CW is a fluorescent stain that binds to the polysaccharide components of the fungal organism’s cell wall. Under ultraviolet light, calcofluor fluoresces, allowing for rapid visualization of cysts as kidney bean-shaped (double parenthesis-shaped or comma-shaped) structures. This stain has been reported to have a sensitivity of 73.8% and a specificity of 99.6% for respiratory specimens (63). The quick turnaround of results makes it a valuable diagnostic tool for PCP, although its requirement for a fluorescent microscope may limit its availability in some laboratories.
- **Toluidine Blue O (TBO) stain:** TBO stain is particularly advantageous due to its ability to highlight cystic forms of *P. jirovecii*. By binding to nucleic acids and certain polysaccharides, it results in a blue coloration of the cysts observable against a contrasting background. TBO’s sensitivity is lower than that of other stains and requires multiple slides to ensure adequate sensitivity. A modified version of TBO, the modified TBO (mTBO), provides cleaner background but the use of toxic reagents, the fact that trophic forms are not visualized, and the overall challenge with interpretation of the stain has decreased the use of TBO and mTBO in most clinical labs. A study involving 231 respiratory specimens demonstrated that the sensitivity of TBO for detecting *P. jirovecii* was 85.7% with a negative predictive value of 99.6% (70). Another study with a cohort of 63 patients reported a 79.6% sensitivity with 100% specificity (29).
- **Grocott’s Methenamine Silver (GMS) stain**: GMS stain, one of the most widely used stains in histology and cytology laboratories, is sensitive for visualizing *P. jirovecii* in lung tissue samples as well as in respiratory secretions. This stain has been reported to have a sensitivity of 79.4% and a specificity of 99.2% for respiratory specimens (63). The GMS stain selectively binds to the polysaccharide components of the cyst wall, staining the cystic forms of the organism dark green or black against a pale background. The appearance of the cysts with this stain is often described as crushed ping pong balls. Its high sensitivity establishes GMS as a gold standard in histopathological evaluations of PCP.
- **Hematoxylin and eosin (H&E) stain:** The H&E stain has been evaluated for visualization of the *Pneumocystis* forms in lung tissues. The hematoxylin dye binds nucleic acids, whereas the eosin targets proteins, resulting in cell nuclei staining blue and cytoplasm staining pink. Since H&E stain does not bind to the organisms’ cell walls, it lacks sufficient sensitivity to detect *Pneumocystis*. However, the H&E stain highlights the frothy intra-alveolar eosinophilic exudate that is characteristic of PCP and with fine focusing, trophic forms may be seen in the exudate. One study developed a protocol for cytocentrifuge respiratory secretions prior to H&E staining to improve sensitivity and demonstrated a sensitivity of 95.7% and a specificity of 100% (71).
- **Papanicolaou’s stain:** Although less sensitive than other stains, Papanicolaou’s stain can be effectively used for cytological samples from lung tissue or BAL specimens. It enhances the contrast of cystic forms, producing characteristic blue or green structures against a pink background for clearer visualization. Its simplicity, widespread use in medical institutions, and effectiveness make it a valuable option in the diagnostic arsenal for PCP. One study with 410 BAL and bronchial wash specimens demonstrated a sensitivity of 83% and a specificity of 100% (72).
- **Periodic acid-schiff (PAS) stain:** The PAS stain is applicable to both lung tissue biopsies and BAL specimens. It targets polysaccharides, producing a bright magenta coloration that distinctly highlights the cyst walls of *P. jirovecii*. The performance characteristics of PAS for the detection of *P. jirovecii* have not been widely reported, but a recent study of 585 patients, including 530 patients with COVID-19, found *P. jirovecii* in 22 patients using PAS, Giemsa, or TBO microscopy. PAS detected *P. jirovecii* in six patients (27.3%) that were negative by Giemsa or TBO (73). The optimal use of PAS is generally achieved when combined with other diagnostic modalities.
- **Giemsa stain:** Giemsa stain is applicable to BAL fluid and lung tissue samples, effectively visualizing both ascus and trophic forms of *P. jirovecii*. It stains nuclei reddish-purple and cytoplasm light blue, providing a comprehensive view of the organism’s life cycle stages. Quick Diff, or Quick Differential Stain, is a rapidly modified Giemsa stain that is also employed to detect *P. jirovecii* with a sensitivity of 49.2% and a specificity of 99.6% for respiratory specimens (63). Additionally, Giemsa’s stains and their derivatives have a sensitivity that may decrease in samples with low organism loads, limiting its utility as a standalone diagnostic tool.

### Immunofluorescent assays

Several studies have shown that immunofluorescent assays provide increased sensitivity compared with direct stains (38, 74). A few immunofluorescent assays, including direct fluorescent assays (DFA) and indirect fluorescent assays (IFA), are commercially available (Table 1). These assays use monoclonal antibodies directed to antigens present on the cell walls of either the ascus or trophic forms of *P. jirovecii*. Positive results are visualized as bright apple-green fluorescence with the characteristic morphologies for *P. jirovecii* forms on a reddish background. The sensitivities and specificities of immunofluorescent assays range from 48%–100% and 81%–100%, depending on the comparator method and the specimen type (75).

**TABLE 1:** Commercially available immunofluorescent tests^*a*^

Vendor (product)^*b*^	Status	Methods	Specimen types	Forms detected	Positive results
Meridian Biosciences (Merifluor *Pneumocystis*)	FDA-IVD	DFA	BAL, BW, induced sputum and unfixed tissues	Ascus (Cysts) Trophozoites Sporozoites	Bright apple-green fluorescence AND characteristic morphologies for *P. jirovecii*
Bio-Rad (MONOFLUO *Pneumocystis jirovecii* IFA)	FDA-IVD	IFA	BAL and sputum	Ascus (Cysts) Trophozoites Sporozoites	
Chemicon, Light Diagnostics (*Pneumocystis carinii* DFA)	CE-IVD	DFA	BAL, sputum, and lung biopsy	Ascus (Cysts) and trophozoites	

^*a*^ FDA-IVD: United States Food and Drug Administration *In vitro* diagnostics; CE-IVD: Conformite Europeene *In vitro* Diagnostics; DFA: Direct Fluorescent Antibody Test; IFA: Indirect Fluorescent Antibody Test; BAL: Bronchoalveolar lavage fluids; BW: Bronchial washings.

^*b*^ Not a complete list.

- **Direct fluorescent antibody (DFA) method**: This technique involves applying labeled antibodies specific to *Pneumocystis* surface glycoproteins to respiratory specimens, such as BAL fluid or lung tissue sections. These antibodies bind directly to the cysts, fluorescing under ultraviolet light and enabling clear visualization of the organism.
- **Indirect fluorescent antibody (IFA) method:** This approach enhances sensitivity by using secondary antibodies conjugated to fluorescent dyes that bind to primary antibodies attached to *Pneumocystis* cysts. This amplification increases the likelihood of detecting low organism levels.

### Nucleic acid amplification tests (NAAT) of respiratory specimens

The advent of NAAT, particularly polymerase chain reaction (PCR), has significantly transformed the detection of *P. jirovecii*, with the first assays developed in the late 1990s (76–78). PCR methods have dramatically improved both the sensitivity and specificity of diagnoses compared with traditional staining techniques, enabling accurate detection in clinical specimens such as BAL fluid, induced sputum, and lung tissue biopsies.

PCR employs specific primers that target conserved regions of the *P. jirovecii* genome (Table 2). The design of these primers is critical; they must be species-specific to minimize cross-reactivity with other organisms. For effective PCR testing, it is crucial to process specimens promptly to minimize DNA degradation. In the case of BAL fluid, low-speed centrifugation can concentrate the organisms, whereas lung tissue samples require homogenization followed by DNA extraction using silica-based columns, magnetic beads, or other methods.

**TABLE 2:** Detection of *P. jirovecii* by PCR^*a*^

Targets	Advantages	Disadvantages	Commercial reagents^*b*^
Dihydrofolate reductase dihydropteroate synthase heat shock protein 70 cdc2 gene mitochondrial ribosomal large-subunit gene	Most sensitive method PCR platforms available in most laboratories Objective interpretation Can be quantitative	No stand-alone FDA-cleared test Does not differentiate between infection and colonization Most expensive method No standardization	ASR: Diasorin, Luminex, Altona, MGB Alert, BioCode, etc. CE-IVD: MycAssay, PneumoGenius, RealStar Pneumocystis, Fungiplex Pneumocystis, Pneumocystis ELITe MGG FDA-IVD: Multiplexed panel: Unyvero LRT BAL

^*a*^ ASR: analyte specific reagents; FDA-IVD: United States Food and Drug Administration *in vitro* diagnostics; CE-IVD: Conformite Europeene *in vitro* diagnostics.

^*b*^ Not a complete list.

Laboratory diagnostics using real-time polymerase PCR (qPCR) have made PCP diagnosis easier and more accurate than the original tests based on conventional, end-point PCR (79). In these assays, a fluorescent probe is used to monitor the amplification process in real time, allowing for qualitative detection and/or quantification of the organism’s load if the assay is conducted with appropriate calibrating quantitative standards. Besides PCR, other NAATs, including loop-mediated isothermal amplification (LAMP), have been developed for the diagnosis of PCP. In one study, LAMP performed on 185 respiratory specimens showed higher PCP DNA detection, 49/185 (26.5%), compared with GMS staining 12/185 (6.5%), and nested PCR 41/185 (22.2%) (80). In another study, LAMP showed a sensitivity of 87.5% when specimens for patients with confirmed PCP were tested (81). LAMP offers the advantage of being conducted without the need for a thermocycler, but these assays are not as widely used in clinical settings for PCP (80, 81).

The significantly higher sensitivity of PCR compared with direct stains and immunofluorescent assays has been reported in several studies (70, 82, 83), with their increased sensitivity, generally above 95% with similarly excellent specificity. PCR has been suggested as the method of choice for PCP (70, 83). However, it is essential to interpret PCR results within the clinical context (70). Although PCR and other qualitative NAATs are highly sensitive, the detection of PCP DNA alone may only reflect colonization and not active disease, leading to overdiagnosis, especially in immunocompromised patients or those with underlying lung conditions (18, 84, 85). To address this challenge, the use of quantitative NAATs has been proposed to not only detect but also quantify PCP DNA concentrations, providing insight into organism burden, which might help differentiate colonization from active infection. In patient populations at risk for PCP, such as HIV-positive individuals or transplant recipients, results of quantitative NAATs have been correlated with symptoms and imaging findings to improve the accuracy of PCP diagnosis. In one study, authors proposed a threshold of 9,655 DNA copies/µL and 12,718 DNA copies/µL in non-HIV and HIV patients, respectively, to distinguish between disease and colonization (86). Maillet and colleagues proposed a cutoff value of 31,600 copies/mL to identify patients with PCP disease (87). Both studies provided a gray zone between the cutoff values for a positive result and a clinically significant positive result. It is important to note, however, that there is currently no standardization across the various laboratory-developed NAATs, either qualitative or quantitative, in terms of the target (single or multi-copy genes), results units (e.g., copies/mL vs copies/µL), the volume of samples and methods used for DNA extraction and amplification. These variables are expected to impact both the sensitivity and specificity of each NAATs for PCP diagnosis. Furthermore, clinical thresholds for distinguishing colonization from disease are not available for any patient population or for any sample type. This remains a major gap in the laboratory diagnosis of PCP. The lack of standardization and efforts to address those issues was recently highlighted in a study by the *Pneumocystis* working group of the Fungal PCR Initiative of the International Society for Human and Animal Mycology (ISHAM) (88). Although these efforts are ongoing, healthcare providers can still improve PCP diagnosis by combining advanced molecular techniques with comprehensive clinical assessment.

### Antigen, antibody testing, and other host biomarkers

The beta-D-glucan (BDG) assay is a valuable tool to support PCP diagnosis, especially in immunocompromised patients. BDG is a polysaccharide component of the fungal cell wall, including PCP, and its presence in serum can indicate invasive fungal infections, including PCP. The test is particularly useful when invasive procedures like bronchoalveolar lavage are not feasible. The FDA-cleared and CE-marked Fungitell assay (Associates of Cape Cod) is a commercially available assay that measures BDG in serum samples (89, 90). This assay utilizes a modified Limulus amebocyte lysate (LAL) derived from the Atlantic horseshoe crab, which specifically reacts with (1→3)-β-D-glucan, resulting in a color change that serves as the readout.

The performance characteristics of BDG testing for PCP diagnosis have been evaluated in several studies. A meta-analysis reported an average sensitivity of 94.8% and a specificity of 86.3%, indicating high accuracy in detecting PCP (91). Using a BDG cutoff level of ≥200 pg/mL in patients with a positive PCR and compatible clinical syndrome, the test achieved a sensitivity of 70%, specificity of 100%, positive predictive value (PPV) of 100%, and negative predictive value (NPV) of 85% (92).

Although elevated levels of BDG can indicate fungal infections, including PCP, it is important to note that false-positive results may occur due to nonfungal factors like surgical gauze, hemodialysis with cellulose membranes, intravenous immune globulin, and specific antibiotics. The serum-based assay is particularly useful as an adjunct to clinical judgment, especially when rapid diagnosis is critical or when traditional diagnostic methods that depend on sputum collection, bronchoscopy, or surgical procedures are not possible. A notable advantage of the BDG assays is their quantitative nature, allowing for correlation with organism burden, disease progression, and therapeutic response. The performance of BDG testing has been evaluated for specimens like BAL, with a pooled sensitivity of 100% for PCP but with poor reproducibility as reported in one meta-analysis and for specimens like cerebrospinal fluid (CSF), although not specifically examining *P. jirovecii* (93, 94). Thus, the clinical utility of BDG in samples other than serum for PCP diagnosis remains an area of investigation.

The Wako β-glucan assay (Wako-BDG; Fujifilm Wako Chemicals) is another test that has received CE marking. This assay also employs the LAL system but measures changes in turbidity, making it compatible with hemolytic, lipemic, or icteric specimens—unlike the Fungitell assay. The results from these assays are analyzed against a calibrated amount of analyte to determine the concentration of BDG, typically reported in picograms per milliliter. Specific cutoff values classify the reactions as positive, negative, or indeterminate. Studies indicate that the Wako β-glucan assay offers sensitivity comparable with that of the Fungitell assay while exhibiting higher specificity (90).

In addition to fungal-derived biomarkers, various host response biomarkers have been explored for diagnosing PCP. Lactate dehydrogenase (LDH) is commonly elevated in patients with PCP and serves as a marker of lung tissue damage (95). Although LDH is non-specific and can be elevated in several lung conditions, it is often used as a supportive indicator alongside other diagnostic methods. Surfactant proteins, particularly SP-A and SP-D, have also been investigated as non-specific potential biomarkers for fungal respiratory infection (96). These proteins play a role in lung homeostasis and surfactant production, and their levels may increase during *P. jirovecii* infection, reflecting alveolar epithelial injury (96). Additionally, enzyme-linked immunosorbent assays (ELISA) targeting the *P. jirovecii* major surface glycoprotein, conducted 3–4 weeks after hospital admission, demonstrated a sensitivity of 63.4%, specificity of 100%, and PPV of 100% for IgG (97). For IgM, the sensitivity, specificity, and PPV were 74.6%, 73.7%, and 89.8%, respectively (97). However, the clinical utility of serology testing may be limited due to high seroprevalence.

## UNMET NEEDS AND EMERGING APPLICATIONS

In 2020, the European Organization for Research and Treatment of Cancer and the Mycoses Study Group Education and Research Consortium (EORTC/MSGERC) released updated definitions of invasive fungal disease (IFD), which included criteria to define *Pneumocystis* (98). A subcommittee of the EORTC/MSGERC, focused on *P. jirovecii* disease in non-HIV patients, provided further guidance which was published in 2021 (99). The applicability of these definitions is expected in clinical, diagnostics, and epidemiological studies in immunocompromised patients without HIV (99). The definitions are based on several criteria including host factors, clinical features, and mycology tests to categorize the disease into probable and proven *Pneumocystis*. From a microbiological standpoint, proven PCP requires the visualization of *P. jirovecii* in tissues or respiratory specimens by direct stains or immunofluorescent assays. For probable PCP, the definition allowed the use of quantitative real-time PCR to detect *P. jirovecii* DNA in respiratory specimens and/or detection of β-D-glucan serum provided that another invasive fungal disease and a false-positive result can be ruled out. Diagnosis of extrapulmonary pneumocystis disease requires microscopic observation of organisms and typically confirmatory NAAT (99).

The complexity of the definitions as described above exemplifies current gaps in the microbiological diagnosis of PCP as recently highlighted by the Fungal Diagnostic Laboratories Consortium (100). For example, to date, only the Unyvero Lower Respiratory Tract (LRT) multiplexed panel (Curetis GmbH, Germany), which is FDA-approved for BAL specimens, includes the detection of *P. jirovecii*. The lack of FDA-cleared, stand-alone *P. jirovecii* specific NAATs limit, the wide adoption of NAATs for PCP in clinical laboratories. Furthermore, the lack of an easy and simple culture system for *in vitro* growth of *P. jirovecii* continues to impair research and development in *P. jirovecii* diagnostics and ultimately the accurate definition of the disease.

A few diagnostic approaches are emerging to improve the diagnostic accuracy of current methods of PCP diagnostics including testing algorithms and detection of cell-free *P. jirovecii* DNA on blood samples. The utility of combining multiple biomarkers of PCP has been evaluated in several studies. Morjaria and colleagues showed that using serum BDG in conjunction with PCP PCR on respiratory samples had the highest positive predictive value for clinically significant PCP in patients with positive PCR and BDG values > 200 pg/mL (92). This approach takes advantage of the high sensitivity of PCR for detection of *P. jirovecii* DNA and the high specificity of BDG for active fungal infections to identify cases of PCP. Other studies have evaluated similar approaches combining BDG results with PCR on nasopharyngeal aspirates (101), serum PCR (102), and plasma cell-free DNA (cfDNA) PCR (103).

The application of real-time PCR on blood samples including plasma and serum as a reliable surrogate sample for the more invasive BAL has recently been evaluated (102–104). The sensitivity and specificity of PCRs for proven PCP ranged between 68%–100% and 70%–100%. The detection of *P. jirovecii* cfDNA by PCR might be associated with more clinically significant PCP than detection in respiratory samples.

PCP treatment and prophylaxis is primarily done using TMP/SMX, a drug that targets two enzymes in *P. jirovecii*, the dihydropteroate synthase (DHPS) and dihydrofolate reductase (DHFR) (105, 106). Concerns for increased resistance have emerged, but antimicrobial susceptibility testing for TMP/SMX against *P. jirovecii* is not currently available, another limitation of the lack of a culture system for this organism and a significant gap in the management of PCP. Molecular approaches have been evaluated to detect the nonsynonymous point mutations in DHPS that result in amino acid substitutions at either or both positions 55 and 57. However, these methods are not commercially available or standardized, and this testing remains an unmet need.

## SUMMARY

Laboratory diagnosis of *P. jirovecii* pneumonia remains challenging as the gold standard of direct detection by microscopic examination (stains or immunofluorescence) has limited sensitivity. Advanced diagnostics methods such as targeted PCR are not commercially available, and laboratory-developed tests are not standardized, limiting comparison across studies. Although a single test might not be sufficient to support the diagnosis of PCP, combining multiple diagnostic tests and taking advantage of each test’s best performance characteristic can result in improved clinical diagnosis of PCP. Further development is urgently needed including the availability of PCP-specific FDA IVD tests besides immunofluorescent assays (e.g., quantitative NAATs and lateral flow PCP antigens tests) for both the diagnosis and management of PCP in immunosuppressed patients.
